# Associations between occupation, leprosy disability and other sociodemographic factors in an endemic area of Brazil

**DOI:** 10.1371/journal.pgph.0000276

**Published:** 2022-09-12

**Authors:** Juan Cisneros, José Antonio Ferreira, Maria Aparecida de Faria Grossi, Thelma de Filippis, Ana Laura Grossi de Oliveira, Sandra Lyon, Jessica K. Fairley

**Affiliations:** 1 Emory University, Atlanta, Georgia, United States of America; 2 Faculdade da Saúde e Ecologia Humana, Vespasiano, MG, Brazil; 3 Faculdade de Medicina, Universidade Federal de Minas Gerais (UFMG), Vespasiano, Brazil; 4 Eduardo de Menezes / FHEMIG, Belo Horizonte, MG, Brazil; 5 Emory University School of Medicine, Atlanta, Georgia, United States of America; Translational Health Science and Technology Institute, INDIA

## Abstract

**Background:**

In Brazil, new leprosy cases with grade-2 disability (G2D) have been increasing. Physical disability has been associated with experienced stigmatization, psychological distress, and social restriction.

**Objectives:**

To identify factors associated with leprosy disability in an endemic area of Brazil focusing on occupational and other sociodemographic factors.

**Methods:**

Between July and December 2015, adult patients with multibacillary leprosy who attended a clinic in Belo Horizonte, Brazil were enrolled. Social, clinical, and demographic factors were collected from an administered questionnaire and medical charts. Occupations were categorized as manual vs non-manual. Descriptive statistics and multivariable logistic regression were performed to study associated factors with disability (Grade 1 disability (G1D) and G2D combined).

**Findings:**

Seventy-three patients were enrolled with 48 (65.8%) presenting with either G1D or G2D at the time of enrollment. Twenty-nine (39.7%) had G2D. About half of the patients (n = 36, 49%) reported a manual labor occupation and reactions were common (n = 53, 73%). On univariate analyses, older age (p = 0.048) and low education (p = 0.007) were associated with disability. On multivariable analyses, only low education (primary or less) was associated with disability (OR = 6.34, 95% CI 1.37, 29.26). Greater distance from clinic, income, smoking, marital status, and occupation were not associated.

**Main conclusions:**

Low education was associated with leprosy disability, consistent with prior studies, and therefore should be a focus for disability reduction programs. While the sample size of this study may have limited detection of associations between disability and social determinants tested, half of the patients reported a manual job, highlighting the need for more extensive studies on associations between occupation, disability, and related injuries.

## Introduction

Leprosy is a result of *Mycobacterium leprae* infection, which affects the skin and peripheral nerves. On occasion, leprosy may even develop in the form of silent neuropathy, allowing *M*. *leprae* bacilli to cause damage long before skin lesions and sensory impairment is noticed. Nerve damage can be irreversible and increases in severity when the infection is left untreated. However, early diagnosis and treatment can prevent permanent nerve damage, consequent disability, and stigmatization that may affect individuals’ health and productivity [1].

The World Health Organization (WHO) has reported an increasing number of cases of leprosy with grade-2 disabilities (G2D) and global leprosy strategies have been focused on reducing the number of new cases with visible deformities since 2005 [2]. In 2015, approximately 210,000 new leprosy cases were detected globally, and 14,000 had G2D, exhibiting an increasing trend from 2006 [3]. In 2019, of the approximately 210,000 new cases, 10, 813 had G2D. This increase and minor decrease in recent years of disability calls for earlier detection methods and identification of factors associated with nerve damage [4]. Of the 138 countries that reported cases, the clear majority emerged from 14 particularly endemic nations.

Brazil has the second highest number of new cases with G2D, accounting for 89% of the new cases with G2D in the Americas [3, 5, 6]. Brazil reported 2,351 new cases with G2D in 2019, an increase over the reported 2,109 in 2019 and the third consecutive year with an increase [4, 7]. Despite the commitment of both local and global organizations, leprosy continues to be a significant cause of morbidity and disability in Brazil, particularly, where disability present at diagnosis has been reported to be increasing in certain states [8]. New case detection rates have reached high levels of endemicity in 18 out of the 27 Brazilian states, including Minas Gerais, with pockets of hyperendemicity (>40 new cases / 100,000 per year) in many areas [9–15].

G2D is usually permanent, leading to noticeable disability, increased stigma, and associated social isolation. Stigma can then significantly impact the patients affecting their physical, psychological, social, and economic well-being [16]. In a hyperendemic region of Brazil, it was reported that functional limitations due to disability had a significant impact on their performance of other activities and social participation long after completing treatment [17]. Moreover, disability has been associated with Functional Activity Limitations (FALs), psychological distress, and social restriction [18–20]. Furthermore, the more disability one has, the higher psychological distress levels, particularly anxiety and depression are more likely to follow [9, 19]. Investigating factors associated with disability will allow for interventions that may reduce the physical impairments, psychosocial burden and improve the overall quality of life for affected patients.

The WHO Global Leprosy Strategy 2016–2020 emphasized new cases with G2D targeting new cases among high-risk and marginalized groups to reduce G2D at diagnosis with the objective to decrease the G2D case rate to <1 per million population [3, 21, 22]. The new strategy has focused to manage leprosy and its complications and prevent new disability, as one of its strategic pillars [23]. Given the challenge of decreasing G2D and the overall high prevalence of disability at diagnosis, investigating risk factors may provide data on at-risk populations that can then be targeted for preventative measures.

Prior studies have evaluated factors such as education, rural residency, medical adherence, marital status, and illiteracy [8, 16, 18, 24–28]. While some clinical factors, such as leprosy reactions, are known to be risk factors for disability, in this study, we investigated the role of select social determinants associated with nerve impairment and disability with a primary focus on occupation and distance between residence and clinic. We hypothesized that manual labor and access to care, defined by the distance from the clinic, are associated with a higher occurrence of grade 1 or grade 2 disability. For those with manual labor occupations, a delay in diagnosis or treatment may result from stigma that may discourage this occupational class from seeking treatment in fear of job loss. Other investigated variables include socioeconomic status, race, education, sex, and select clinical factors.

## Materials and methods

### Study site and population

Between July and December 2015, adult cases (18 years and older) of multibacillary (MB) leprosy who presented for care to a dermatologic reference center in Belo Horizonte, Minas Gerais, Brazil, were enrolled in a study to study risk factors for leprosy reactions [29]. Eligible participants included patients with multibacillary (MB) disease as defined by the World Health Organization (WHO), with some cases of borderline tuberculoid (BT) (5 or more skin lesions), and all cases of borderline borderline (BB), borderline lepromatous (BL) and lepromatous (LL). Paucibacillary (PB) cases were excluded, given low rates of disability. Patients could be at any stage in their treatment–newly diagnosed, on multi-drug therapy (MDT), or following MDT [1]. Those with and without leprosy reactions (Type 1 and Type 2) were included.

### Data collection

An in-person questionnaire was administered by study staff, and included questions on demographic topics such as race, marital status, occupation, socioeconomic status, residence (urban vs. rural district), education level, and smoking status. Data abstracted from the medical records included the leprosy type per Ridley Jopling categorization [30], date of original diagnosis, and the presence of disability (Grade 1 or Grade 2) at the time of diagnosis or first appointment at the clinic (if data on disability not available from diagnosis). Disability grade was based on the WHO disability grading system [31] (Table 1).

**Table 1:** WHO disability criteria (adapted from Johannes) [31].

WHO disability grading	
Hands and Feet	Grade 0: no anasthesia, visible deformity or damage
	Grade 1: anaesthesia present, no visible deformity or damage
	Grade 2: visible deformity or damage present
Eyes	Grade 0: no eye problem due to leprosy; no evidence of visual loss
	Grade 1: eye problems due to leprosy but vision not severely affected
	Grade 2: severe visual impairment

Manual labor was defined as occupations that rely heavily on physical work and intense strength such as farmers, day laborers, bricklayers, handymen, and fishermen. Those who worked in a less intensive physical environment were grouped as non-manual laborers and included office clerks, housekeepers, and taxi drivers. These assignments follow the standard form of occupational segmentations in current literature and in the Brazilian National Classification of Economic Activities [18, 32, 33]. Residence demographics were obtained by the distinction between rural/urban on the questionnaire and through measurement of distance in kilometers from the reference clinic to their municipality of residence. Far and near was defined by using the geographical distribution of the data set, with an average of 150 km for the distance to the clinic being determined as the cutoff. Both the median and mean ranged between 139–150 km, respectively, so we thought that 150 km would be a meaningful cutoff to analyze distance from clinic. Furthermore, that would equate to about a 2–3 hour drive by car, which is a moderate distance considering the range from within the same city to 569 km distant. The distance was taken from the reference clinic location to the residence city center. The low-income variable was defined as those earning less than 1X the minimum wage versus those earning more and low education was defined as having received no more than primary education. Primary education is the second stage of education in Brazil for 6 to 14-year-old students. Older age was defined as 60 years of age or older. This categorization is consistent with other studies on leprosy disability [9, 34].

### Data analysis

Descriptive statistics and univariate analyses were performed on the main study variables and p-values were calculated to compare those patients with or without disability (Grade 1 or Grade 2 combined) using chi-square, Fisher’s exact test, or t-test where appropriate. A p-value of <0.05 was taken as statistically significant. For this paper, we performed a post-hoc analysis to look at this research question of disability, understanding that the original question was looking at risk factors for leprosy reactions [29]. The sample size (48 cases of disability, 25 without disability) was sufficient to detect about an odds ratio of 4.5 assuming that about 15–18% of controls would report being a manual laborer (as per a prior study [27]) and using a power of 0.80 and alpha of 0.05. Given the exploratory nature and secondary data analysis, a larger sample size was not feasible to detect a smaller difference between the two groups in respect to the primary exposure of manual labor. Multivariable logistic regression was performed to calculate adjusted odds ratios on the main study variables. The model included disability (either Grade 1 or Grade 2) as the outcome and manual labor as the primary exposure with covariates including distance to clinic, income, low education (8^th^ grade or less), marital status, age, sex, and race. The model was evaluated for collinearity (by measuring condition indexes), interactions, and confounding. Interactions between the main exposure, hard labor, and each of the other variables were assessed by the chunk test. A change in estimates approach was utilized to determine confounding and variables were dropped from the model if the inclusion of them did not change the point estimate of the main exposure by more than 10% or did not improve the primary measure’s precision. All analyses were done using SAS v9.4 (Carey, NC), Tableau 10.3 (Seattle, WA), and ESRI ArcMap 10.6 (Redlands, CA).

### Ethics

Ethical approval was obtained from the institutional review boards of Emory University and Faculdade da Saúde e Ecologia Humana (FASEH). Ethical approval was also granted by the Institutional Review Board at Hospital Eduardo de Menezes. All participants provided written informed consent.

### Inclusivity in global research

Additional information regarding the ethical, cultural, and scientific considerations specific to inclusivity in global research is included in the Supporting Information (S1 Text).

## Results

**Fig 1:**
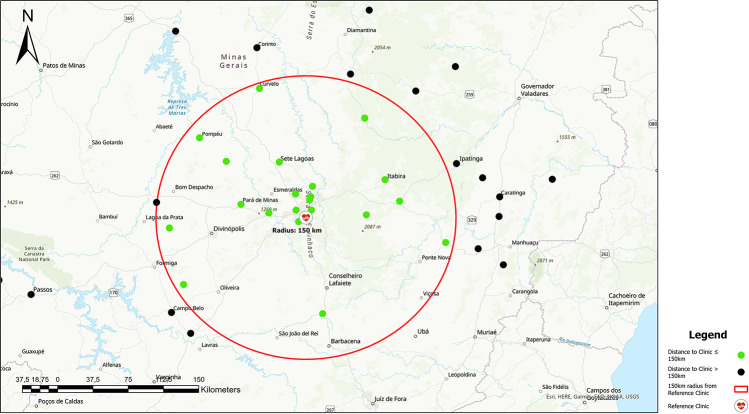
Map of the Brazilian state of Minas Gerais and capital city, Belo Horizonte, patient residences mapped, circle delineating 150km from reference clinic, green markers within radius (ESRI ArcMap).

Of the seventy-three patients enrolled, 65.8% (n = 48) had evidence of disability at the time of either diagnosis or first clinic visit with 39.7% (n = 29) presenting with G2D, 26% (n = 19) with G1D, and 34.2% (n = 25) without disability. About a quarter was female (27.4%, n = 20). The mean age was 51.2 years (±14.3) and a quarter of them were above the age of 60 (n = 18, 24.7%). Of the total study population, the majority were nonwhite (76.1%, n = 53, Table 2). Despite less than half of the patients residing in a rural location (29.2%, n = 14), almost half of the total study population (45%, n = 32) lived greater than 150 km away from the reference clinic. This range of distance varied from 0 km to 574 km with an average of 151 km, with twelve patients living within the city of Belo Horizonte but many residing at the perimeter of the state (Fig 1). The monthly income was on average above the minimum wage, with only twenty patients overall earning below this benchmark. Most of our patients had received primary education or less (83.3%, n = 60) and 44 of them had a disability. Low education was associated with disability on the univariate analysis (P = 0.007) as was age as a continuous variable (p = 0.048). Residence, income, smoking, marital class (married vs not), and occupation were not associated with disability on univariate analysis (Table 2).

**Table 2:** Main demographic and clinical variables of study population, comparing those with and without disability on univariate analysis, using chi-square unless otherwise noted. Bolded p-values represent significance at a p-value <0.05.

Variable	Disability (n = 48)	No Disability (n = 25)	Total (n = 73)	P-value
**Age, years (mean, SD)**	53.6 (14.2)	46.7 (13.6)	51.2 (14.3)	**0.048** *
**Age > 60 years**	14 (29.2)	4 (16.0)	18 (24.7)	0.22^
**Gender, n (%)**				
**Female**	13 (27.1)	7 (28.0)	20 (27.4)	0.93
**Grade of Disability, n (%)**		N/A	N/A	---
**Grade 1**	19 (39.6)			
**Grade 2**	29 (60.4)			
**Clinical form of leprosy, n (%)**				
**Borderline tuberculoid**	6 (12.5)	4 (16.0)	10 (13.7)	0.88
**Borderline borderline**	14 (29.2)	8 (32.0)	22 (30.1)	
**Borderline lepromatous**	4 (8.3)	1 (4.0)	5 (6.9)	
**Lepromatous**	24 (50.0)	12 (48.0)	36 (49.3)	
**Reaction present, n (%)**	36 (75.0)	17 (68.0)	53 (72.6)	0.52
**Race, n (%) (1 miss)**				
**Nonwhite**	35 (72.9)	18 (75.0)	53 (73.6)	0.85
**White**	13 (27.1)	6 (25.0)	19 (26.4)	
**Residence, n (%)**				
**Rural**	14 (29.2)	4 (16.0)	18 (24.7)	0.26^
**>150 km from clinic**	21 (44.7)	11 (44.0)	32 (44.4)	0.93
**(1miss)**				
**Monthly income, n (%)**				
**< 1 times the minimum wage**	16 (33.3)	4 (16.0)	20 (27.4)	0.12^
**Smoking, n (%) (1 miss)**				
**Yes**	16 (33.3)	4 (16.7)	20 (27.8)	0.17^
**Occupational class, n (%)**				
**Manual labor**	25 (52.1)	11 (44.0)	36 (49.3)	0.56
**Marital Status, n (%)**				
**Married**	27 (56.3)	14 (56.0)	41 (56.2)	0.98
**Primary education or less, n (%)**	44 (91.7)	16 (64.0)	60 (82.2)	**0.007**

*P-value determined by t-test

^P-value determined by Fisher’s exact test.

Table 3 shows the multivariable logistic regression with variables left in the model after tests of collinearity, interaction, and confounding. The multivariable analysis results show that primary education or less was strongly associated with disability at an odds ratio of 6.34 (95% CI 1.37, 29.26). Patients aged 60 years or older had an odds ratio of disability of 2.10 (95% CI 0.47, 9.47), but the results were not statistically significant, controlling for other factors. The presence of leprosy reaction, rural residence, manual labor, and distance to clinic >150 km was also not significantly associated with disability (Table 3).

**Table 3:** Unadjusted and adjusted odds ratio of factors associated with disability (Grade 1 or 2) left in the final model. Bolded values are significant at an alpha of <0.05. *Defined as either Type 1 or Type 2 leprosy reaction.

Variable	Unadjusted OR	95% CI	p-value	Adjusted OR	95% CI	p-value
**Manual labor**	1.34	0.50, 3.60	0.56	0.69	0.21, 2.26	0.54
**>150 km from clinic**	0.96	0.36, 2.56	0.93	0.60	0.18, 1.96	0.40
**Primary education or less**	**5.50**	**1.46, 20.79**	**0.007**	**6.34**	**1.37, 29.26**	0.02*
**Age ≥ 60 years**	2.16	0.63, 7.45	0.22	2.10	0.47, 9.47	0.33
**Presence of “reaction”**	1.41	0.49, 4.09	0.53	2.09	0.63, 6.94	0.23
**Rural residence**	2.16	0.63, 7.45	0.22	2.22	0.52, 9.51	0.28

## Discussion

In our study, we found a high proportion of patients with the MB leprosy who had grade 1 or grade 2 disability. While we did not limit our assessment to patients presenting at diagnosis, 2/3 (n = 48) of study participants had objective evidence of nerve impairment at the time of the study. Of those with apparent neuropathy, 60% (n = 29) had the most severe, G2D (Table 2). This is consistent with other studies, which show a large proportion of G2D cases at diagnosis in Brazil [24]. Of those with disability in our study, 3/4 were male (n = 35, 72.8%), similar to reported findings across Brazil [14, 24, 35–37].

The most significant finding in our study was the association between low education (primary or less) and higher occurrence of Grade 1 or 2 disability, with an adjusted odds ratio of 6.34 (95%CI 1.37, 29.26), consistent with other studies [18, 24, 28, 38, 39]. A higher education level often translates to increased health literacy and it is, therefore, not surprising that it acts as a protective factor for disability [34]. With a greater education level, patients may be more informed, have access to care, present to medical care earlier, and avoid nerve function impairment.

While our study did not show an association of disability with manual labor, those with such occupation may be at greater risk of accidental damage while working with disability [40]. Of all the sociodemographic factors, few have looked at occupational roles and types. Occupational studies in Brazil investigating leprosy have considered traditional social factors, such as the human development index and the proportion of people living in households, but none have specifically studied occupation types [34]. However, studies from Southern Asia have shown that occupations categorized as manual work have a significant association with nerve impairments, and manual laborers have significantly more leprosy disability than those with non-manual occupations [27, 41]. Our results showed no association between manual labor and disability, in contradiction to the findings of Withington, Sarkar, et al. [27]. Identifying occupational types with nerve damage could lead to directed treatment strategies of patients with higher risk of disability-related injuries. More than half of our participants had an occupational class defined as manual labor (53%), similar to other studies which have measured this variable. The fact that we did not find associations between disability and manual labor may be due to the small sample size of this study and the fact that there was a considerable amount of disability found in the study. In addition, the actual work performed by the participants may have been varied and perhaps not generalizable across contexts and countries.

Distance to reference clinic as a measure of access to care was also not associated with disability. Access to care was a hard measure to define and absolute distance may not have been an adequate measurement of access. A drive-time area map and a more detailed investigation of transportation barriers may provide a deeper understanding of accessibility. Access to care is likely an under-recognized social determinant of health in regard to leprosy leading to potential treatment delays and further disability.

Lastly, other studies have found that certain age groups are associated with disability [24, 28, 38] and have reported a trend of increased impairment with increased age [26, 27, 41]. In univariate analysis, age (as a continuous variable) was associated with disability but age was not associated with disability on the multivariable analysis. This may be due to overall similar ages of patients in this study.

Limitations of this analysis are the cross-sectional nature of the analysis and small sample size, which is often the case with leprosy given the overall rarity of the disease even in endemic areas. Since the analysis is one-point in time, causation is not possible to assess. The original scope of the study was to investigate the association of leprosy reactions with co-infections [29] but given the large burden of disability in this study sample, we chose to do an exploratory analysis of social determinants of health and disability. While the sample size was only adequate for a finding of a strong association between our primary exposure of manual labor and disability (predicted OR of 6), which we did not find, it was large enough to show the association of education and disability, (at a power of 80% and alpha of 0.05), attesting to the validity of the association in addition to the literature supporting this finding. For further study on occupation, larger samples sizes are needed. In addition, this clinic is a reference center for more severe cases and the degree of disability found may have also limited the delineation of associated factors. Lastly, stigma is an unmeasured confounder in this study and it is difficult to know how that contributed to disability in these participants.

However, our most important finding of educational links to disability supports the need for increased awareness and health campaigns to bring people to care and ensure they understand how to lessen their risk of permanent nerve damage. Also, the high proportion of low education, manual labor, and low socioeconomic status of these patients point to potential health disparities of people with leprosy in Brazil and call for more research and program implementation to address these issues.

## Supporting information

S1 Text Questionnaire on inclusivity in global research. (DOCX)

S2 Text Patient questionnaire. (DOCX)

## References

[1] Cross H. The prevention of disability for people affected by leprosy: whose attitude needs to change? Lepr Rev 2007 Dec;78(4):321–329. 18309705

[2] World Health Organization. Weekly Epidemiological Record (WER), Global leprosy situation. World Health Organization 2012 Aug 24,;87.

[3] World Health Organization Department of Control of Neglected Tropical Diseases. WHO | Global leprosy update, 2015: time for action, accountability and inclusion. WHO 2016 Sep 2.

[4] World Health Organization. Weekly epidemiological record, Global leprosy (Hansen disease) update, 2019: time to step-up prevention initiatives. World Health Organization 2020 Sept 4,:417–440.

[5] Alberts CJ, Smith WCS, Meima A, Wang L, Richardus JH. Potential effect of the World Health Organization’s 2011–2015 global leprosy strategy on the prevalence of grade 2 disability: a trend analysis. Bull World Health Organ 2011 -7-01;89(7):487–495. doi: 10.2471/BLT.10.085662 21734762 PMC3127268

[6] Kerr-Pontes LRS, Barreto ML, Evangelista CMN, Rodrigues LC, Heukelbach J, Feldmeier H. Socioeconomic, environmental, and behavioural risk factors for leprosy in North-east Brazil: results of a case-control study. Int J Epidemiol 2006 Aug;35(4):994–1000. doi: 10.1093/ije/dyl072 16645029

[7] World Health Organization Department of Control of Neglected Tropical Diseases. WHO | Global leprosy update, 2016: accelerating reduction of disease burden. WHO 2017 Sep 1:501–520.

[8] Cabral-Miranda W, Chiaravalloti Neto F, Barrozo LV. Socio-economic and environmental effects influencing the development of leprosy in Bahia, north-eastern Brazil. Trop Med Int Health 2014 Dec;19(12):1504–1514. doi: 10.1111/tmi.12389 25244417

[9] Leekassa R, Bizuneh E, Alem A. Prevalence of mental distress in the outpatient clinic of a specialized leprosy hospital. Addis Ababa, Ethiopia, 2002. Lepr Rev 2004 Dec;75(4):367–375. 15682974

[10] Monteiro LD, Alencar CH, Barbosa JC, Novaes, Candice Cristiane Barros Santana, Silva, Rita de Cássia Pereira da, Heukelbach J. Limited activity and social participation after hospital discharge from leprosy treatment in a hyperendemic area in north Brazil. Revista Brasileira de Epidemiologia 2014;17:91–104. doi: 10.1590/1415-790x201400010008eng 24896785

[11] Castro S, Pereira J, Santos P, Abreu G, Oliveira V, Luciane F, et al. Leprosy incidence, characterization of cases and correlation with household and cases variables of the Brazilian states in 2010 *. An Bras Dermatol 2016;91:28–33. doi: 10.1590/abd1806-4841.20164360 26982775 PMC4782643

[12] Lana FCF, Lanza FM, Velásquez—Meléndez G, Branco AC, Teixeira S, Malaquias LCC. Leprosy distribution, by gender, in the Municipality of Governador Valadares, Minas Gerais, Brazil. Hansenologia Internationalis (Online) 2003 00/;28(2):131–137.

[13] Nicchio MVC, Araujo S, Martins LC, Pinheiro AV, Pereira DC, Borges A, et al. Spatial and temporal epidemiology of Mycobacterium leprae infection among leprosy patients and household contacts of an endemic region in Southeast Brazil. Acta Trop 2016 Nov;163:38–45. doi: 10.1016/j.actatropica.2016.07.019 27469619

[14] Lana FCF, Fabri, Angélica da Conceição Oliveira Coelho, Lopes FN, Carvalho APM, Lanza FM. Deformities due to Leprosy in Children under Fifteen Years Old as an Indicator of Quality of the Leprosy Control Programme in Brazilian Municipalities. Journal of Tropical Medicine 2013 /03/19;2013:e812793. doi: 10.1155/2013/812793 23577038 PMC3614053

[15] Ministry of Health of Brazil. Rate of Leprosy detection by state and region, Brazil from 1990–2016, per 100,000 inhabitants. 2017; Available at: http://portalarquivos2.saude.gov.br/images/pdf/2017/julho/10/Taxa-de-detec—-o-geral-de-hansen—ase-1990a2016-.pdf. Accessed Jan 14, 2019.

[16] Murto C, Chammartin F, Schwarz K, da Costa M, Kaplan C, Heukelbach J. Patterns of Migration and Risks Associated with Leprosy among Migrants in Maranhão, Brazil. PLoS neglected tropical diseases 2013 Sep 1,;7(9). 10.1371/journal.pntd.0002422 PMC3764227 24040433

[17] Regional Office for South-East Asia, World,Health Organization. Global Leprosy Strategy 2016–2020. Accelerating towards a leprosy-free world. Monitoring and Evaluation Guide. New Delhi: World Health Organization. Regional Office for South-East Asia; 2017.

[18] de Castro LE, da Cunha AJ, Fontana AP, Halfoun VL, Gomes MK. Physical disability and social participation in patients affected by leprosy after discontinuation of multidrug therapy. Lepr Rev 2014 Sep;85(3):208–217. 25509722

[19] Reis FJJ, Lopes D, Rodrigues J, Gosling AP, Gomes MK. Psychological distress and quality of life in leprosy patients with neuropathic pain. Leprosy Review 2014 /09/01;85(3):186–194. 25509719

[20] van Brakel WH, Sihombing B, Djarir H, Beise K, Kusumawardhani L, Yulihane R, et al. Disability in people affected by leprosy: the role of impairment, activity, social participation, stigma and discrimination. Glob Health Action 2012 -7-20;5. doi: 10.3402/gha.v5i0.18394 22826694 PMC3402069

[21] World Health Organization. WHO | Accelerating progress on HIV, tuberculosis, malaria, hepatitis and neglected tropical diseases, A new agenda for 2016–2030. 2015; Available at: http://www.who.int/about/structure/organigram/htm/progress-hiv-tb-malaria-ntd/en/. Accessed Jan 13, 2019.

[22] WHO | Department of control of neglected tropical diseases. WHO | Ending the neglect to attain the Sustainable Development Goals: A road map for neglected tropical diseases 2021–2030. 2020; Available at: http://www.who.int/neglected_diseases/resources/who-ucn-ntd-2020.01/en/. Accessed July 14, 2020.

[23] WHO Control of Neglected Tropical Diseases. Towards zero leprosy Global Leprosy (Hansen’s disease) Strategy 2021–2030. World Health Organization 2021 April 15,:1–30.

[24] Monteiro LD, Martins-Melo FR, Brito AL, Alencar CH, Heukelbach J. Physical disabilities at diagnosis of leprosy in a hyperendemic area of Brazil: trends and associated factors. Lepr Rev 2015 Sep;86(3):240–250. 26665359

[25] Silva DRX, Ignotti E, Souza-Santos R, Hacon SdS. Hanseníase, condições sociais e desmatamento na Amazônia brasileira. Revista panamericana de salud pública 2010 Apr;27(4):268–275. doi: 10.1590/s1020-49892010000400005 20512229

[26] Kumar A, Girdhar A, Girdhar BK. Risk of developing disability in pre and post-multidrug therapy treatment among multibacillary leprosy: Agra MB Cohort study. BMJ Open 2012;2(2):e000361. doi: 10.1136/bmjopen-2011-000361 22454186 PMC3330256

[27] Withington SG, Joha S, Baird D, Brink M, Brink J. Assessing socio-economic factors in relation to stigmatization, impairment status, and selection for socio-economic rehabilitation: a 1-year cohort of new leprosy cases in north Bangladesh. Lepr Rev 2003 Jun;74(2):120–132. 12862253

[28] Santos VS, Matos AM, de Oliveira LS, de Lemos LM, Gurgel RQ, Reis FP, et al. Clinical variables associated with disability in leprosy cases in northeast Brazil. The Journal of Infection in Developing Countries 2015 /03/02;9(03):232–238. doi: 10.3855/jidc.5341 25771459

[29] Fairley JK, Ferreira JA, de Oliveira ALG, de Filippis T, Grossi MA, Chaves LP, et al. The Burden of Helminth Coinfections and Micronutrient Deficiencies in Patients with and without Leprosy Reactions: A Pilot Study in Minas Gerais, Brazil. Am J Trop Med Hyg 2019 11;101(5):1058–1065. doi: 10.4269/ajtmh.18-0502 31549606 PMC6838598

[30] Ridley DS, Jopling WH. A classification of leprosy for research purposes. Lepr Rev 1962 Apr;33:119–128. doi: 10.5935/0305-7518.19620014 14492126

[31] Brandsma JW, Van Brakel WH. WHO disability grading: operational definitions. Lepr Rev 2003 Dec;74(4):366–373. 14750582

[32] IBGE, Comissão Nacional de Classificação. National Classification of Economic Activities—CNAE v 2.2. 2.2nd ed. Rio de Janeiro, Brasil: IBGE; Comissão Nacional de Classificação (Brasil); 2015.

[33] Melchior H, Velema J. A comparison of the Screening Activity Limitation and Safety Awareness (SALSA) scale to objective hand function assessments. Disabil Rehabil 2011;33(21–22):2044–2052. doi: 10.3109/09638288.2011.560328 21955054

[34] Santos VS, Oliveira LS, Castro FDN, Gois-Santos V, Lemos LMD, Ribeiro, Maria do C. O., et al. Functional Activity Limitation and Quality of Life of Leprosy Cases in an Endemic Area in Northeastern Brazil. PLOS Neglected Tropical Diseases 2015;9(7):e0003900. doi: 10.1371/journal.pntd.0003900 26132166 PMC4489006

[35] Varkevisser CM, Lever P, Alubo O, Burathoki K, Idawani C, Moreira TMA, et al. Gender and leprosy: case studies in Indonesia, Nigeria, Nepal and Brazil. Lepr Rev 2009 Mar;80(1):65–76. 19472853

[36] Henry M, GalAn N, Teasdale K, Prado R, Amar H, Rays MS, et al. Factors Contributing to the Delay in Diagnosis and Continued Transmission of Leprosy in Brazil–An Explorative, Quantitative, Questionnaire Based Study. PLOS Neglected Tropical Diseases 2016;10(3):e0004542. doi: 10.1371/journal.pntd.0004542 26977811 PMC4792453

[37] Alves CJM, Barreto JA, Fogagnolo L, Contin LA, Nassif PW. Evaluation of the degree of incapacity of patients with a diagnosis of leprosy at a dermatology service in the state of São Paulo. Revista da Sociedade Brasileira de Medicina Tropical 2010 08/;43(4):460–461. 20802951 10.1590/s0037-86822010000400025

[38] Moschioni C, Antunes, Carlos Maurício de Figueiredo, Grossi MAF, Lambertucci JR. Risk factors for physical disability at diagnosis of 19,283 new cases of leprosy. Revista da Sociedade Brasileira de Medicina Tropical 2010 02/;43(1):19–22. doi: 10.1590/s0037-86822010000100005 20305962

[39] Ponnighaus JM, Fine PE, Sterne JA, Malema SS, Bliss L, Wilson RJ. Extended schooling and good housing conditions are associated with reduced risk of leprosy in rural Malawi. Int J Lepr Other Mycobact Dis 1994 Sep;62(3):345–352. 7963906

[40] Nardi SMT, Ikehara E, Pedro HSP, Paschoal VDA. Characterization of the profession/occupation of individuals affected by leprosy and the relationship with limitations in professional activities. Indian J Lepr 2012 Jan-Mar;84(1):1–8. 23077777

[41] Sarkar J, Dasgupta A, Dutt D. Disability among new leprosy patients, an issue of concern: an institution based study in an endemic district for leprosy in the state of West Bengal, India. Indian J Dermatol Venereol Leprol 2012 May-Jun;78(3):328–334. doi: 10.4103/0378-6323.95449 22565433

