# Supplementary material for: Associations between occupation, leprosy disability and other sociodemographic factors in an endemic area of Brazil
Source: PLOS Glob Public Health. 2022 Sep 12;2(9):e0000276. doi: 10.1371/journal.pgph.0000276 (PMC10021318; doi:10.1371/journal.pgph.0000276)

**Title:** **Parasitic co-infections and select micronutrient deficiencies may be risk factors for the development of reactions in multibacillary cases of Hansen’s disease.**

**PARTICIPANT DEMOGRAPHIC SURVEY**

INTERVIEWER: PLEASE READ ANY TEXT IN ITALICS PRIOR TO EACH QUESTION AS WELL AS THE QUESTION ITSELF. READ THE ANSWERS ONLY WHEN INSTRUCTED. NEVER READ “DON’T KNOW” OR “REFUSED TO ANSWER” AS AN ANSWER. PLEASE READ THE INTRODUCTION IN ITALICS BELOW TO ALL PARTICIPANTS. IF THERE IS AN ARROW FOLLOWED BY A QUESTION NUMBER AFTER A RESPONSE, PLEASE SKIP TO THAT QUESTION. CIRCLE ALL RESPONSES UNLESS THERE IS A PLACE TO ENTER A NUMBER OR AN ANSWER TO AN OPEN-ENDED QUESTION.

| *Hello, my name is_______________________. Thank you for participating in this study. I am part of a team of scientists from FASEH and Emory University who are investigating the association of parasitic infections, nutritional deficiencies and the risk of developing Hansen’s disease reactions. Your contribution will provide valuable knowledge that may help prevent complications of patients with Hansen’s disease. You have signed a consent form and to remind you, everything you say here will be kept private. Your participation is completely voluntary and you may change your mind at any point. Your name will not be mentioned in any presentation/publication related to this study. However, we do appreciate your involvement. The expected duration of this survey is 5-10 minutes and will involve basic questions about you and your household. Do you still agree to participate?*  *YES NO* |
| --- |

**Interview start time ___:___ am/pm**

| Demographic and Household Information  Question | Responses |
| --- | --- |
| 1. What is the city / state do you live? | _______________________________________________ |
| 2. Is it urban or rural? | 1. Urban  2. Rural |
| 3. This site is a coverage area of the health centers? | Yes………………………….1  No……………………………2  Don’t know……………….99 |
| 1. How old were you, in years, on your last birthday? | [___ ___ ___]  Refused……………….98  Don’t know………….99 |
| 1. Gender | Male ……………………..1  Female…………………..2 |
| 1. How many people live in your home? | [___ ___]  Refused…………...…..98  Don’t know………….99 |
| 1. How would you describe your race? | Fill in:___________________________________ |
| 1. What is your marital status? | Single..........................................................0  Single, but living with a partner…..1  Married......................................................2  Separated..................................................3  Divorced....................................................4  Widowed...................................................5  Other (please specify).......................68  Refused to respond………………..….98 |
| 1. Do you currently have a job? | Yes, full-time out of the house……....1  Yes, part-time out of the house …….2  Yes, full-time from home………...……3  Yes, part-time from home…………….4  No ………………………………………………5  Refused to respond…………………….98 |
| 1. Is your permanent residence in the state of Minas Gerais? | Yes………………………. 1 🡪 Go to 7  No…………………………2 🡪 Go to 6  Refused………..……..98  Don’t know……..…..99 |
| 1. What state do you live in? | [_____________________________________]  Refused………..……..98  Don’t know……..…..99 |
| 1. What municipality do you live in? | [_____________________________________]  Refused………..……..98  Don’t know……..…..99 |
| 1. What town or village do you live in? | [_____________________________________]  Refused………..……..98  Don’t know……..…..99 |
| 1. Please list any other states or municipalities you have lived in the past. | Other states __________________________________  _________________________________________________  Other municipalities_________________________  _________________________________________________ |
| 1. Please tell me whether this household has the following items (circle): | Refrigerator......................................1  Television.............................................2  Working Automobile.......................3  VCR/DVD Player ..............................4  Household Phone..............................5  Mobile Phone .....................................6  Air Conditioner ..................................7  Refused………..……..98  Don’t know……..…..99 |
| 1. What is your average household income per month? | SR________________________  Refused ……..….……98 |
| 1. How many children does that income support? | [___ ___ ]  Refused ……………..98 |
| 1. How many children aged 17 years or younger are living in this household? | [___ ___]  Refused………..……..98  Don’t know……..…..99 |
| 1. How do you assess the material status of your family? *Pick the most appropriate statement* | We can easily satisfy our needs…………………………….…..………………...1  We can somewhat satisfy our needs………………..................................................2  We can hardly satisfy our needs (make ends meet)…………………………………………………..3 |
| 1. What is your highest educational level attained? | None …………………………………………..……………0  Fundamental education incomplete (1-7+) ....1  Fundamental education complete (8)................2  Intermediate education incomplete (9-11) .....3  Intermediate education complete (11) ..............4  Technical level................................................................5  Any higher education..................................................6  Post-graduate.................................................................7  Refused..........................................................................98  Don’t know…………………………………………..…..99 |

PART 2: Parasitic Infections

*I will now ask some questions about parasitic infections.*

21. Do you remember any parasitic infection that was diagnosed after the diagnosis of Hansen’s disease?

1. Yes, since I was diagnosed with Hansen’s disease

2. Yes, before I was diagnosed with Hansen’s disease

3. No, never

98. I do not know

88. Refused to answer

22. If so, you could point out the possible cause?

1. Ascaris

2. Shistosoma

3. Hookworm

4. Tape worms

5. Pin worm

6. Ameoba

7. Giardia

8. Cannot remember the name

9. Other (specify): ___________________

98. I do not know

88. Refused to answer

23. Were you ever treated for this parasitic infection?

1. Yes

2. No

3. Don’t know

4. Refused to answer

24. What is your regular source of water? Please select from the following options:

1. Running water

2. pond or river

3. Stormwater

4. Pit or cistern

68. Other (please specify): ___________________

98. I do not know

25. Do you treat or filter the water to drink?

1. Yes

2. Do not

98. I do not know

26. How is the sewage system of your home? Please choose from the following answers:

1. Sewage piped

2. Sewage without plumbing

3. There is no sewer system.

4. Latrine

68. Other (please specify): _______________

98. I do not know

27. Do you wash all your fruits and vegetables before cooking and serving?

1. Yes

2. No

98. I do not know

28. How do you wash your vegetables?

1. Wash them in tap water

2. Wash them in water with vinaegar

3. Wash them with filtered water and low concentration of bleach

4. I don’t wash them

98. I do not know

88. refuses to answer

29. Is there a pet in your home?

1. Yes

2. No

98. I do not know

30. Do you perform any type of work activity and / or recreational contact with water from rivers, lakes, stream, wells and or other natural water source?

1. Yes

2. No

68. Other (please specify): ___________________

98. I do not know

Part 3:

NUTRITION

Food Consumption Frequency Questionnaire

1. Have you changed your food intake or diet recently?
2. No
3. Yes, to lose weight
4. Yes, for medical reasons
5. Yes, to be a vegetarian or reduce meat intake
6. Yes, to reduce salt intake
7. Yes, to reduce cholesterol
8. Yes, to gain weight
9. Other ____________________________
10. Are you taking any dietary supplements?
11. No,
12. Yes, regularly
13. Yes, but not regulary

If yes, which supplement (s)

___________________________ _______________________________ ______________________________


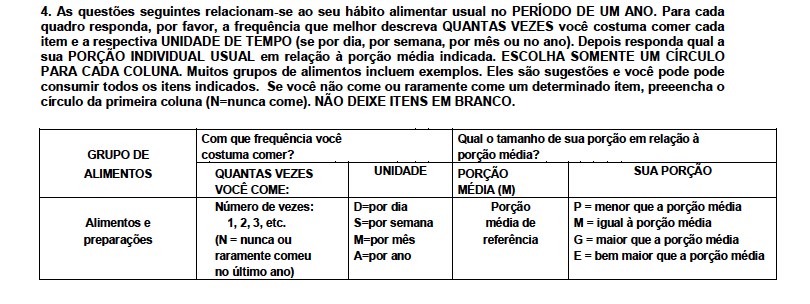


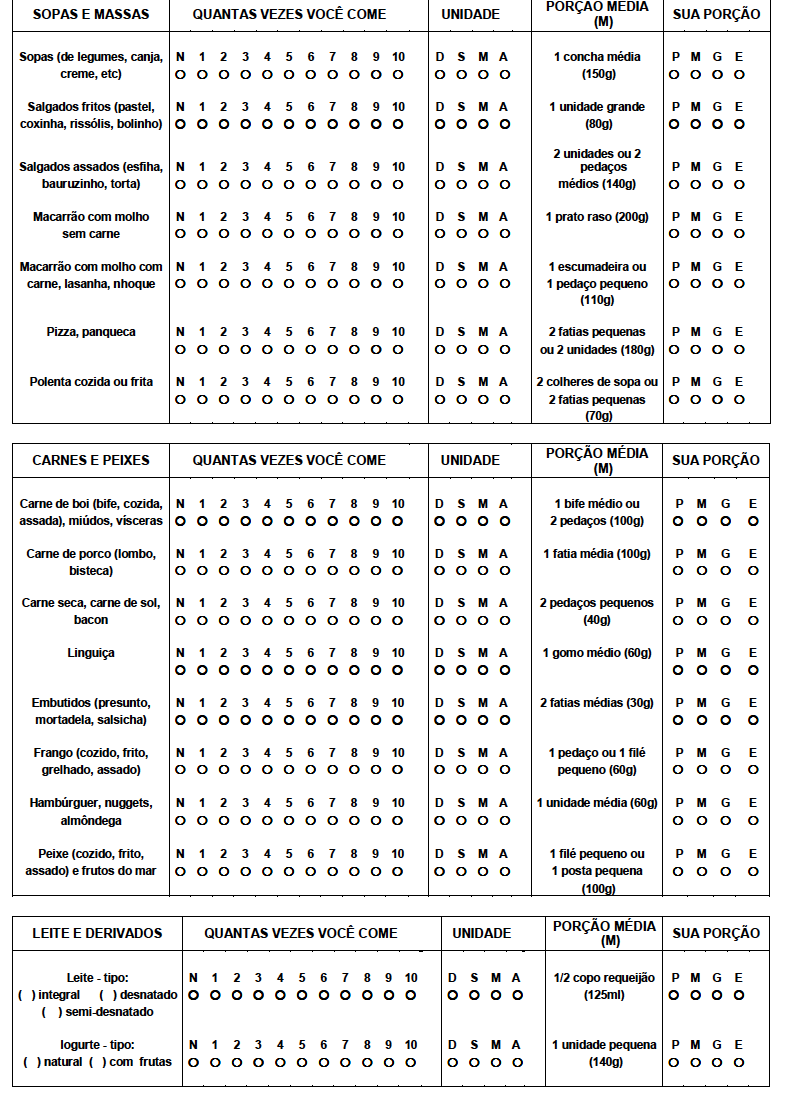


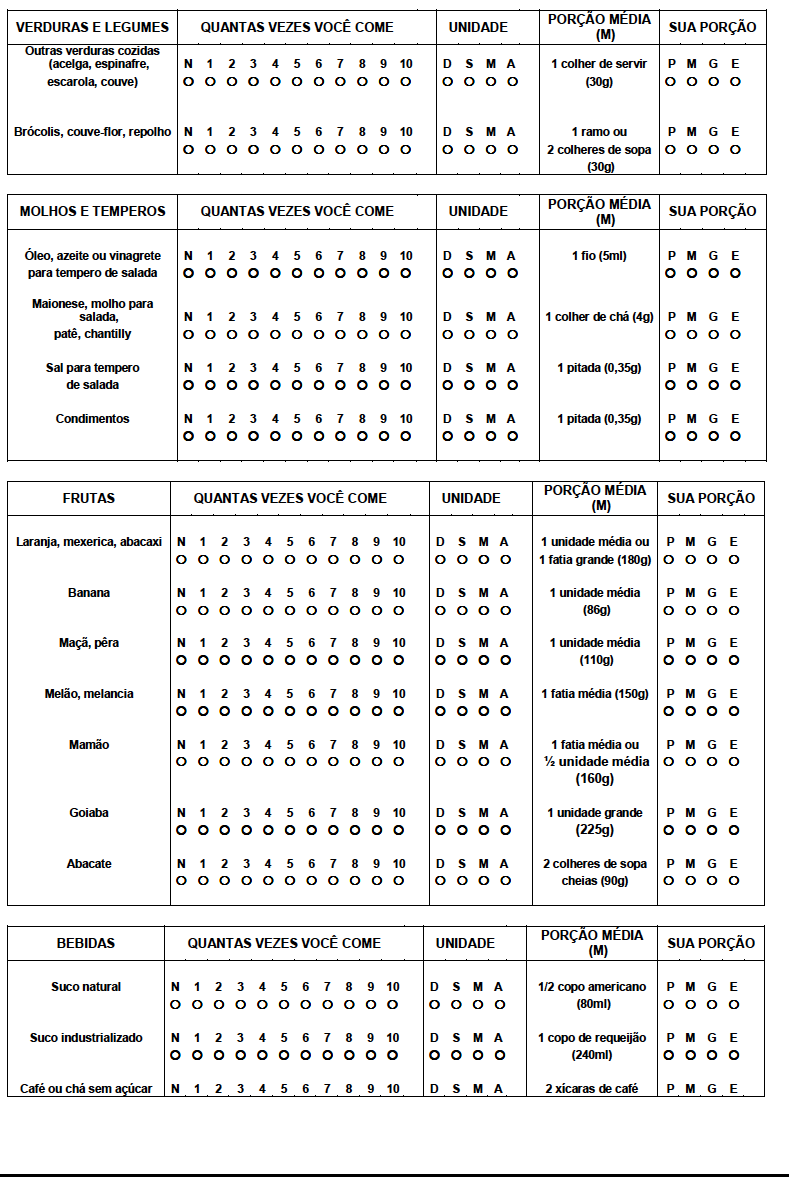

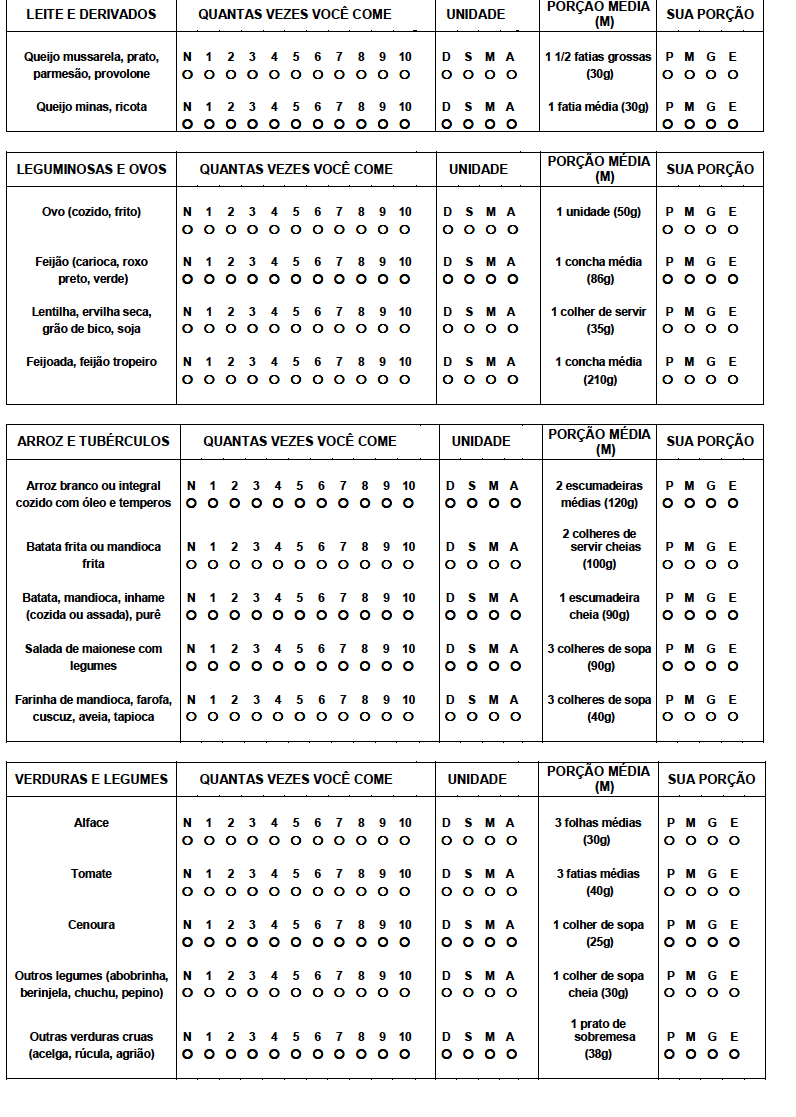


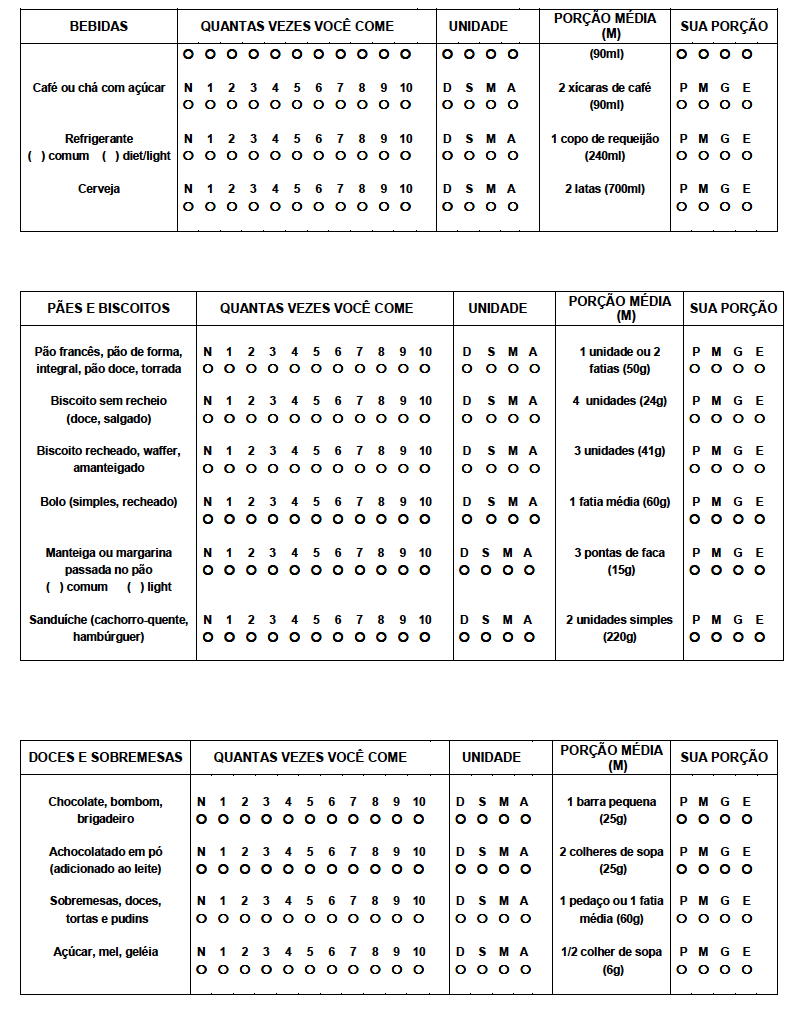


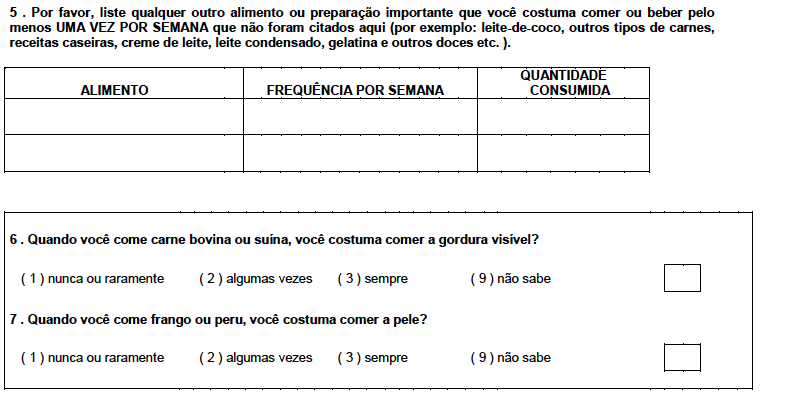

Supplement: S2 Text — (DOCX) [file pgph.0000276.s002.docx]
